# The Role of Infection and Immune Responsiveness in a Case of Treatment-Resistant Pediatric Bipolar Disorder

**DOI:** 10.3389/fpsyt.2017.00078

**Published:** 2017-05-22

**Authors:** Rosalie Greenberg

**Affiliations:** ^1^Medical Arts Psychotherapy Associates, P.A., Summit, NJ, United States

**Keywords:** pediatric bipolar disorder, treatment resistance, inflammation, infections and psychiatric illness, molecular mimicry

## Abstract

A case of psychotropic-resistant pediatric bipolar disorder is presented. Both awareness and proper treatment of previously unrecognized infections and their effects on the immune system were very important in stabilizing the patient’s psychiatric symptoms.

## Introduction

There is a growing recognition that the immune system plays an important role in the development and perpetuation of certain neuropsychiatric disorders (1, 2). Increased awareness of factors such as inflammation and autoimmunity or immune hyperresponsiveness may facilitate new understanding of mental disorders and potentially provide new insight into treatment-resistant patients. This case report provides an example of a possible link between pediatric bipolar disorder and immune-mediated processes. The presentation is intended to inform future discussions of novel treatment interventions targeted to neuron inflammatory pathways.

## Background

### Identifying Information

P, a 16-year-old high school student who resides with his parents and two younger siblings, ages 13 and 11 years, has been in treatment with his present psychiatrist since the age of 10 years. Upon initial presentation, history was positive for longstanding difficulty with mood shifts, oppositional behavior, and verbal and physical aggressive outbursts that have been refractory to multiple medication trials.

### History of Present Illness

Despite the product of an unremarkable pregnancy and delivery, his parents felt he was somewhat different from birth. He did not like being held and was hard to soothe. Once ambulatory, P appeared very curious, overactive, and frequently into “mischief.” In toddlerhood, he also began to exhibit difficulty with moodiness and oppositional defiant behaviors with peers as well as authority figures. He could be extra silly, but more often easily unhappy and angry. In nursery school, he would hit and bite peers when frustrated and was defiant with teachers. Behavioral interventions were successful.

**Figure 1:**
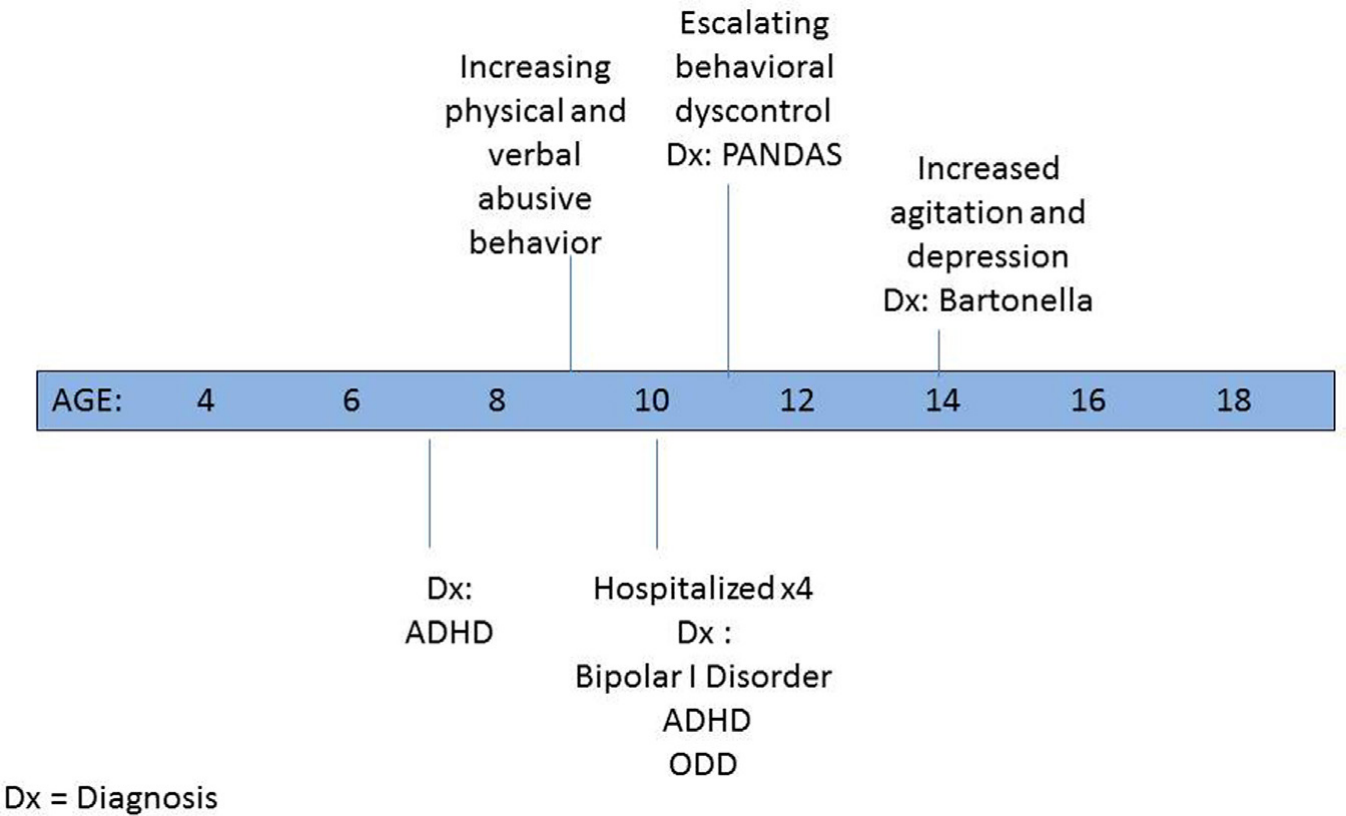
**Psychiatric timeline (in years)**.

By the time he was in the first grade, since his interactions were fraught with conflict, following evaluation, he was deemed eligible for special services in the academic setting. P was given a 1:1 aide both in school to help keep him on task and on the bus to prevent behavioral outbursts. Psychological counseling was initiated at the age of 7 years and soon thereafter a psychiatrist placed him on medication for a newly diagnosed attention-deficit hyperactivity disorder (ADHD) (3) (see Figure 1). By the age of 10 years, despite multiple medication trials and family and individual psychotherapy, P’s behavior escalated to the point that he was verbally and physically violent and uncontrollable at home. Between ages 10 and 11 years, he was psychiatrically hospitalized for more than a week on four occasions because of his aggressive threats and actions. He repeatedly stated that he wanted to kill his family and himself. Discharge diagnoses included bipolar I disorder—mixed, ADHD—combined type, and oppositional defiant disorder.

P’s behavior appeared treatment resistant with minimal improvement despite multiple medication trials prior to and during hospitalizations. These included dexmethylphenidate hydrochloride extended-release tablets, methylphenidate hydrochloride extended-release tablets in a variety of preparations, mixed dextroamphetamine salts in extended release, risperidone, fluoxetine, and aripiprazole. Parents noted that he became more angry and agitated when he was on the stimulants. In an attempt to reevaluate his situation, soon after hospital discharge all psychotropic medication was stopped.

Over a 6-week period, post medication discontinuation, he started to become less volatile and violent, but exhibited rapid cycling behaviors such as laughing a lot, talking fast, exhibited increased energy, suddenly became very interested in cleaning his room, had problems falling asleep, and became preoccupied with and talked about Star Wars incessantly. He would then shift to periods of anger and destructive behavior and verbalize that he felt everything was wrong. Initiation of carbamazepine and low-dose olanzapine decreased the rapid cycling and eliminated the violent physical behavior, but his negative and critical speech at home continued. He did minimal schoolwork, was socially withdrawn, and described by mother as joyless, rarely smiling, and verbalized being unhappy. His appetite was poor. He said he hated most food, and although he grew taller he had not gained weight in 2 years.

### Past Medical History

He was hospitalized for 48 h at 4 days old due to a low-grade fever (99°) of unknown origin.

### Family History

#### Psychiatric History

Maternal side: bipolar disorder in at least three generations, postpartum depression, alcoholism, obsessive-compulsive disorder, anger management issues.
Paternal side: strongly positive for alcoholism.

#### Immune Dysfunction

Maternal side: mother diagnosed with chronic fatigue syndrome; Hashimoto’s thyroiditis in a first cousin; thyroid dysfunction of unclear etiology in another relative.
Paternal side: a paternal grandfather with aplastic anemia.

Both parents had relatives with adult-onset diabetes mellitus.

## Course of Treatment

A change in antipsychotic to risperidone and titration of his carbamazepine dose helped decrease the frequency of his aggressive outbursts temporarily. Of note, intermittently over the years in treatment, P experienced transient episodes of urinary frequency and urgency despite a negative urine culture. In addition, he occasionally exhibited transient motor tics (eye blinking, mouth opening, and a shoulder shrug).

After 8 months, his mood and behavior worsened and residential treatment was considered. Laboratory testing looking for evidence of a possible infectious trigger was done. Streptococcal and mycoplasma titers were drawn even though his illness was chronic, not acute in onset as seen in pediatric autoimmune neuropsychiatric disorders associated with streptococcal infections (PANDAS) or pediatric acute-onset neuropsychiatric syndrome (PANS) (4). Antistreptolysin O and anti-DNAase B titers were markedly elevated at 790 (normal 0–200) and 1,340 (normal 0–170). Throat culture was negative. His pediatrician prescribed a 20-day course of amoxicillin and clavulanate potassium. Psychiatric medication remained unchanged. Within a few days, P’s agitation lessened and he became happier, more affectionate, and more engaged in his schoolwork. Upon antibiotic discontinuance, his irritability returned, and resumption of treatment helped him regain control. After 6 weeks of treatment, repeat ASO was 791 with an anti-DNAase B titer of 1,090, i.e., basically unchanged. A Cunningham panel to check for evidence of immune dysfunction and antineural antibodies was unremarkable (5). A lithium trial was unsuccessful. Given his resumption of his behavioral problems soon after the completion of the second antibiotic trial, an immunology consult with a PANDAS/PANS specialist was obtained.

Consultation resulted in the diagnosis of autoimmune encephalopathy as well as an active sinus infection. P was placed on amoxicillin and clavulanate potassium, azithromycin, and a brief course of prednisone starting at 30 mg and tapered over 1 week. When next seen here, 3–4 weeks after initiating treatment, there was a dramatic positive change in P’s functioning.

P’s bossy and irritable behavior would intermittently return when a family member or a peer became ill. The addition of ibuprofen 300 mg bid often helped him become less aggressive but still annoying. If these symptoms continued and escalated, a change in his antibiotics was often efficacious. Occasional use of a brief steroid trial also appeared beneficial.

In the middle of eighth grade, P became severely angry and depressed and developed hyperacusis. Evidence of a *Bartonella* infection on fluorescent–*in situ* hybridization (FISH) testing was found and subsequently treated (6). Psychiatric symptoms lessened, and his noise sensitivity dissipated. Maintenance on risperidone and carbamazepine continued throughout treatment.

Currently, P is functioning fairly well outside the home and his bossiness and negativity with his family are nowhere near where they were at initial presentation. He remains on his psychiatric medication, along with amoxicillin and clavulanate potassium for streptococcal prophylaxis.

## Discussion

Although this case of childhood bipolar disorder has a strong genetic component, it also provides some support for the hypothesis that the various psychiatric symptoms (that are consistent with bipolar disease) may have been exacerbated by abnormal neuro-immune responses initiated by systemic infections. In this article, the relevant infections being group A beta-hemolytic *Streptococcus* and *Bartonella*. Despite intense antibiotic treatment, it took approximately 3 months for the ASO titers to begin to decrease. This ongoing evidence of immune activation could be seen as consistent with Bechter and Mueller’s hypothesis that psychiatric symptoms may be the result of a low-level “smoldering” inflammatory process in the central nervous system (CNS) or what could be viewed as “a chronic mild encephalitis or encephalitic process” (7). Younger and Bouboulis speculated that the sustained high streptococcal titers as occurred in this case could to be the result of reinfection, or slower rates of the decline in antibody rise as well as possibly a more potent immune response (8).

Currently, inflammation in mood disorders is an active area of exploration (9–11). Anti-inflammatory agents such as acetylsalicylic acid, nonsteroidal anti-inflammatory agents (e.g., ibuprofen), minocycline, and cox-2 inhibitors have been studied as adjuvants to bolster the effects of treatment in adults with depression (12). Some caution has been raised about the combination of ibuprofen and selective serotonin reuptake inhibitors as there is some evidence of increased risk of gastrointestinal adverse reactions (13). The use of ibuprofen in P’s case appeared to be very helpful especially when he was exposed to other children with illness. The recrudescence of his symptoms can be postulated to be the result of a hyperimmune response to stimulation (other children illness) that was modified and somewhat contained by ibuprofen’s anti-inflammatory action. Care was taken to monitor for any evidence of possible increased bleeding, i.e., increased bruising, nosebleeds, and hematuria. Use of ibuprofen was also time limited (i.e., generally not more than a few days or weeks at a time), and complete blood counts, bleeding parameters, and renal and hepatic function tests were monitored if there was any indication of a problem.

The recurrent positive response of psychiatric symptomatology to brief steroid trials lends support to the hypothesis that their anti-inflammatory actions were helpful in intervening in his extreme agitation. In this case, it helped P to control his violent and destructive behavior as opposed to more common use of steroids for out-of-control physical symptoms.

Although these infections may seem temporally related to the patients’ difficulties that does not mean causality. On a few occasions when antibiotics were stopped, P’s functioning quickly deteriorated with responsiveness upon resumption of the antibiotic medications. This raises the questions of whether or not there was reinfection, or perhaps a remnant of the previously treated infection perpetuating ongoing antibody production? Current maintenance on antibiotic treatment is used to minimize future exposure to infectious triggers that could potentially reactivate the immune system and cause an augmented autoimmune response. For P, this immune hyperstimulation could result in another psychiatric symptom flare-up.

An important question in the interconnections of infections, immune dysfunction, autoimmunity, and psychiatric illness is how does the peripheral immune system penetrate or communicate with the CNS which is an area protected by the blood–brain barrier (BBB). Of note, P was first diagnosed with a sinus infection when he was initially seen by the immunologist at the age of 11 years and he experienced intermittent recurrences. The existence of any sinusitis prior to the first visit is unknown. The high preponderance of sinus infections in bipolar youth has been previously noted by this author in a previous text (14).

There is some evidence that nasal lymphoid tissue may play a role in communication between the peripheral immune system and immune activity in the CNS. Dileepan et al. found that reinocculating mouse nasal lymphoid tissue with group A *Streptococcus* (GAS) resulted in “GAS-specific Th17 cells” migrating “into the brain through the cribriform plate along olfactory sensory axons and induce BBB breakdown and IgG extravasation” (15). This is a significant finding as the investigators were able to find GAS-specific TH17 cells in tonsils of individuals naturally exposed to GAS. Whether or not the human nasal lymphoid tissue is part of the path to the brain in the development of some autoimmune illnesses (especially those with neuropsychiatric manifestations) needs more investigation.

P did not have PANDAS or PANS due to the chronic nature of his illness. The overlap of several features seen in youth with PANDAS/PANS and pediatric bipolar disorder has been reported previously (16). P exhibited some symptoms that are seen in youngsters with the former named disorders including intermittent urinary symptoms, transient motor tics, and sudden onset of hyperacusis. These symptoms may be related to the immune dysfunction and part of a mild encephalitic process. Further exploration of the mechanism of initiation and perpetuation of these symptoms is needed. An additional commonality between the two groupings of disorders is the significant number of individuals with immune disturbances in P’s pedigree and the heightened number of individuals with immune disturbances in the family histories of PANDAS patients (17).

The concept of molecular mimicry in which the mimicry of host antigens by infectious agents may cause cross-reactive autoimmune responses to epitopes within host proteins has been used to explain autoimmune dysfunction in some illnesses (e.g., PANDAS, post-viral myocarditis) (18). The Cunningham Panel whose purpose is to measure “the level of circulating antibodies directed against antigens concentrated in the brain, and measures the ability of these and other autoantibodies to increase the activity of an enzyme (CaMKII) that upregulates neurotransmitter in the brain” was unremarkable in this case (5). This test focuses on four possible autoantibodies directed against specific neuronal antigens, including: dopamine D1 receptor, dopamine D2L receptor, lysoganglioside GM1, and tubulin. An unremarkable result may be because other significant neural autoantibodies are not checked in this testing panel.

There are a number of different species of the Gram-negative bacteria *Bartonella* that can cause illness in animals and human populations, but it has been considered more of an opportunistic type of infection (19). Three types are responsible for most of the *Bartonella* infections seen in people: *Bartonella henselae* (Cat Scratch Fever), *Bartonella quintana* (Trench fever), and *Bartonella* Bacilliformi (Carrion’s disease). In P’s case, FISH testing identified the pathogen as *Bartonella* but it is not set up to identify the individual species (6).

In humans, *Bartonella* infections are associated with a variety of neuropsychiatric manifestations and can affect small blood vessels in the CNS. Symptoms attributed to Bartonellosis can include: mild cognitive impairment; white matter “subcortical disconnection”; impaired executive functioning; working memory impairment; processing speed delay; mood lability; severe agitation; panic disorder and treatment-resistant depression (20, 21). A number of autoimmune diseases have been known to be associated with Bartonellosis including autoimmune thyroiditis, Systemic Juvenile Rheumatoid Arthritis, vasculitis, glomerulonephritis, autoimmune hemolytic anemia, transverse myelitis, Henoch Schonlein Purpura, and Guillain–Barré Syndrome (22, 23).

## Conclusion

This case illustrates how the interplay of infections and subsequent aberrant immune responses contributed to the manifestations of childhood-onset bipolar disorder in one youngster. It also raises the question of what is the meaning treatment resistance? Could it be that we are not looking at the correct causalities or all the relevant factors involved in the individual case? Although genetic, infectious, and immunologic influences are important factors in the development of psychiatric illness, the significance of epigenetic and psychosocial factors (peer groups, education attained, family functioning) should not be underestimated. Future controlled research may be warranted to elucidate the nature of the interrelationship of pediatric bipolar symptomatology, infectious illness, and immune/autoimmune responses.

## Ethics Statement

Case report contains disguised identifying data and has been written up with approval from the family.

## Author Contributions

The author confirms being the sole contributor of this work and approved it for publication.

## Conflict of Interest Statement

The author declares that the research was conducted in the absence of any commercial or financial relationships that could be construed as a potential conflict of interest.
